# Supplementary figures and images for: Phenolic Profiles, Antioxidant, and Hypoglycemic Activities of Ribes meyeri Fruits
Source: Foods. 2023 Jun 18;12(12):2406. doi: 10.3390/foods12122406 (PMC10297579; doi:10.3390/foods12122406)

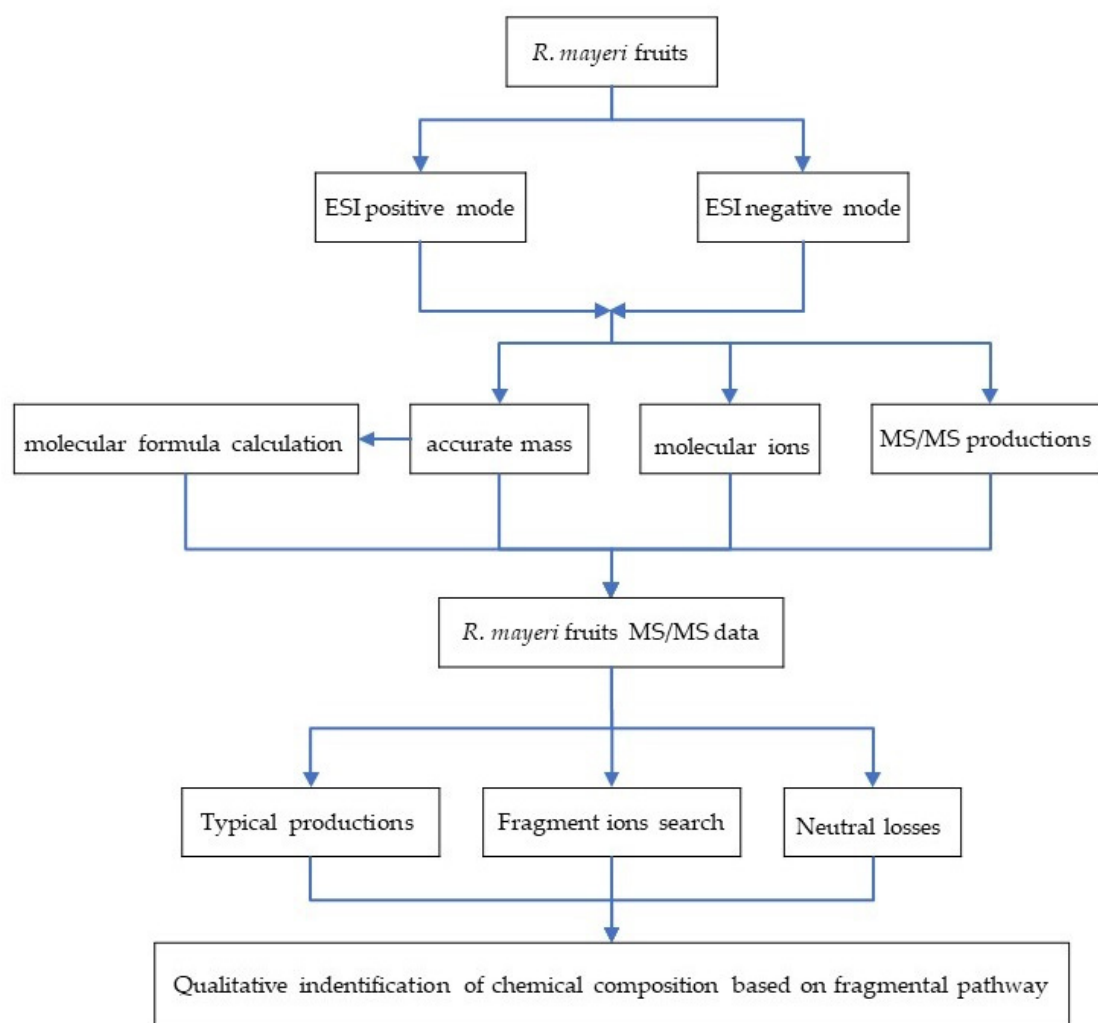

**Figure S1.** Compound identification scheme of the research methods.

Supplement: Supplementary file 1 [file foods-12-02406-s001.zip › foods-2398065-supplementary material.pdf]
